# Priming of NK Cell Anti-Viral Effector Mechanisms by Direct Recognition of Human Cytomegalovirus

**DOI:** 10.3389/fimmu.2013.00040

**Published:** 2013-02-21

**Authors:** Aura Muntasell, Marcel Costa-Garcia, Andrea Vera, Noemí Marina-Garcia, Carsten J. Kirschning, Miguel López-Botet

**Affiliations:** ^1^Hospital del Mar Medical Research InstituteBarcelona, Spain; ^2^Immunology Unit, Pompeu Fabra UniversityBarcelona, Spain; ^3^Institute of Medical Microbiology, University of Duisburg-EssenEssen, Germany

**Keywords:** NK cells, HCMV, pathogen-associated pattern recognition receptors

## Abstract

Natural killer (NK) cells play an important role in the defense against viral infections. Activation of resting NK cells is tightly controlled by the balance of surface inhibitory and activating receptors and aided by cytokines released by accessory cells along the anti-viral response. On the other hand, NK cells express functional pattern recognition receptors (PRRs) whose function has been mostly addressed by the use of synthetic agonists. The present study was undertaken to investigate whether NK cells could directly recognize a complex pathogen such as Human Cytomegalovirus (HCMV). Exposure of primary human NK cells to HCMV (TB40/E strain) induced the expression of CD69, promoted IFNγ secretion, and increased their cytotoxic activity against HCMV-infected autologous monocyte-derived dendritic cells. The divergent response induced by infective and UV-inactivated virions indicated the involvement of different NK cell sensors in the recognition of HCMV. The fact that NK cell activation could be partially prevented by blocking mAb specific for IFNAR and TLR2, together with the induction of IFNβ mRNA, supported the involvement of IFNβ and TLR2 in the response to HCMV. Thus, our data indicate that simultaneous activation of several PRRs leads to the autonomous priming of NK cell effector functions and could be a previously unappreciated mechanism presumably contributing to the control of HCMV infection.

## Introduction

Natural killer (NK) cells provide an important first line of defense against viral pathogens. NK cells participate in the viral clearance by lysing infected cells and through the production of IFNγ and TNFα which exert a non-cytolytic control of the infection and modulate the induction of protective specific responses. Activation of NK cells is tightly controlled by the balance between germ-line encoded inhibitory and activating receptors (Lanier, 2008) and modulated by the action of innate cytokines (Biron et al., 1999). More recently, a role for pathogen-associated pattern recognition receptors (PRRs) as regulators of the NK cell biology has emerged. Initial studies based on mRNA detection on human NK cell extracts, described TLR1 as the highest expressed, followed by moderate levels of TLR2, TLR3, TLR5, and TLR6, and low levels of TLR4, TLR7, TLR8, and TLR9 mRNA (Hornung et al., 2002; Chalifour et al., 2004; Hart et al., 2005; Gorski et al., 2006; Marcenaro et al., 2008). TLR2, TLR3, TLR7, and TLR9 have been shown to be intracellular in human NK cells (Roda et al., 2005; Eriksson et al., 2006; Girart et al., 2007; Marcenaro et al., 2008), although surface expression has also been reported for TLR2 and TLR3 (Becker et al., 2003; Hart et al., 2005). Generally, exposure of primary NK cells to individual synthetic Toll-like receptor (TLR) agonists did not enhance NK cell effector functions unless combined with cytokines such as IL-12 or type I IFNs, yet conflicting data can also be found (Sivori et al., 2004; Hart et al., 2005; Roda et al., 2005; Eriksson et al., 2006; Girart et al., 2007; Duluc et al., 2009). Activation of TLR2, TLR7/8 promotes the secretion of IFNγ while having little effect in NK cell cytotoxic responses (Hart et al., 2005; Girart et al., 2007); in contrast, TLR9 signaling induces the expression of activation markers and enhances Ab-dependent cytotoxicity (Hornung et al., 2002; Sivori et al., 2004; Roda et al., 2005; Girart et al., 2007). One of the strongest agonist for NK cell function is polyI:C which in combination with IL-2 or IL-12 promotes both IFNγ and cytotoxic responses through the secretion of IFNβ (Sivori et al., 2004; Hart et al., 2005; Girart et al., 2007; Duluc et al., 2009). Recently, sensing of poly I:C in NK cells has been shown to mostly depend on RIG-1-like receptors (Perrot et al., 2010), introducing cytosolic PRRs in the regulation of NK cell biology. Indeed, NK cells express functional NOD-2, NRLP3, Mda5, and RIG-1(Athie-Morales et al., 2008; Duluc et al., 2009; Perrot et al., 2010).

To date, few studies have addressed the consequences of pathogen sensing by NK cells. Interaction of human NK cells with *Mycobacterium bovis* involved TLR2 and triggered NK cell activation (Marcenaro et al., 2008). NK cells have been shown to exhibit direct microbicidal activity against *Cryptococcus neoformans* (Marr et al., 2009). Remarkably, direct recognition of vaccinia virus by TLR2 in NK cells was necessary for the efficient control of this infection in a murine model, highlighting the importance of NK cell pathogen recognition *in vivo* (Martinez et al., 2010).

The contribution of NK cells in the immune response to viruses is evidenced by the fact that patients with NK cell deficiencies are susceptible to recurrent herpesviral infections, including Human Cytomegalovirus (HCMV; Biron et al., 1989; Orange and Ballas, 2006). HCMV is an enveloped dsDNA virus which *in vitro* can productively infect fibroblasts and, less efficiently, endothelial, and differentiated myelomonocytic cells (Mocarski and Courcelle, 2001). Cytomegalovirus infection activates the innate immune system through both TLR-dependent and -independent pathways. Mice strains harboring specific deletions evidenced the participation of TLR2 and TLR9 in the recognition of MCMV envelope glycoproteins and viral DNA, respectively (Krug et al., 2004; Tabeta et al., 2004; Szomolanyi-Tsuda et al., 2006; Zucchini et al., 2008). TLR3 and TLR7 are also involved in sensing CMV (Tabeta et al., 2004; Zucchini et al., 2008) although it is not clear whether they are activated during initial infection or later during the viral replication cycle. In humans, HCMV glycoproteins gB and gH activate TLR2 signaling (Boehme et al., 2006). CMV also stimulates cytosolic DNA sensors such as DAI/ZBP1 in human fibroblasts (DeFilippis et al., 2010) and the AIM2 inflammasome along MCMV infection (Rathinam et al., 2010). Epidemiological data also supports the involvement of TLR2 and TLR9 in HCMV sensing. Three genetic studies have identified specific single nucleotide polymorphisms in TLR2 and TLR9 genes which are highly predictive of susceptibility to HCMV infection/reactivation in adult recipients of liver and allogeneic stem cell transplants, respectively (Kijpittayarit et al., 2007; Carvalho et al., 2009; Kang et al., 2012).

The present study was undertaken to investigate the consequences of the direct interaction between NK cells and a complex pathogen such as HCMV. We provide data supporting the capacity of NK cells to directly sense HCMV, identifying some of the mechanisms underlying this process, and dissecting the consequences of this interaction on NK cell function. Our results support that the simultaneous engagement of different PRRs on NK cells by HCMV leads to an accessory cell-independent enhancement of NK cell effector mechanisms, likely contributing to the development of an efficient response against the infection.

## Materials and Methods

### Antibodies and flow cytometry analysis

FACS analysis was performed using monoclonal antibodies specific for the following surface molecules: CD56-allophycocyanin (APC), CD3-Phycoerythrin (PE), CD19-PE, CD123-PE, CD14-PE, CD1a-PE, CD69-PE, IFNγ-PE, CD107a-fluorescein isothiocyanate (FITC; BD Biosciences Pharmingen, San Diego, CA, USA). Anti-NKp30 (clone AZ20), and -NKp46 (clone BAB281) mAbs were kindly provided by Prof. A. Moretta (University of Genova, Italy). MAb anti-CD16 (clone KD1) has been previously described. Cells were pretreated with human aggregated IgG (10 μg/ml) to block Fc receptors, and subsequently labeled with specific antibodies. Cell viability was measured using the FITC Annexin V Apoptosis Detection Kit II *(*BD Pharmingen, San Diego, CA, USA) according to the manufacturer’s instructions.

For blocking experiments the following monoclonal antibodies were used at saturating concentrations: antagonistic mAb specific for TLR2 (clone T2.5; Meng et al., 2004) was kindly provided by Dr. Carsten Kirschning (Institute of Medical Microbiology, University of Duisburg-Essen, Essen, Germany). Blocking antibody against IFN receptor chain 2 (IFNAR; clone MMHAR-2, IgG2a) was from Calbiochem. An anti-myc mAb (9E10, IgG1) was used as negative control.

### NK cell isolation

PBMC were obtained from heparinized blood samples by separation on Ficoll-Hypaque gradient (Lymphoprep; Axis-Shield PoC AS, Oslo, Norway). Samples were obtained with the informed consent of the subjects and the study protocol was approved by the institutional Ethics Committee (CEIC-IMAS). NK cell purification was performed by negative selection using EasySep Human NK Cell Enrichment kit (StemCell Technologies, Grenoble, France) according to the manufacturer’s recommendations.

### HCMV preparation and NK cell treatment

Purified stocks of HCMV TB40/E strain (Sinzger et al., 1999; kindly provided by Dr. Christian Sinzger, Institute for Medical Virology, University of Tubingen, Tubingen, Germany) were prepared as previously described (Muntasell et al., 2010). Briefly, MRC-5 cells were infected at 0.1 moi and supernatants recovered when maximum cytopathic effect was reached. Virus was pelleted twice through a sorbitol cushion (20% d-sorbitol in TBS (25 mM Tris-HCl, pH7.4, 137 mM NaCl) by centrifugation 90 min at 27,000 × *g* at 15°C, resuspended in serum-free DMEM, and titered by standard plaque assays on MRC-5 cells. Inactivation of viral stocks was achieved by UV-light using an UV-crosslinker (Biorad GS genelinker UV chamber). A fraction of viral stocks was passed through 0.1 μm filter to eliminate viral particles. Viral UV-light inactivation and the absence of infectious virus in filtered viral stocks were confirmed by treating the MRC-5 fibroblast cell line with the viral preparations, followed by detection of the viral IE-1/IE-2 antigen with a mouse anti-CMV mAb (clone mab810, Millipore). No infected cells could be detected in MRC-5 monolayers incubated with UV-inactivated TB40/E or filtered viral stocks. In contrast, IE-1 nuclear staining was observed in 100% of MRC-5 cells treated with TB40/E.

Isolated NK cells were cultured in complete RPMI medium supplemented with 200 μ/ml rhIL-2 (unless noted in the figure) in the presence or absence of HCMV TB40/E strain (moi 5–10, based on NK cell number), and CD69 expression was monitored after 24 h by flow cytometry. For inhibition of TLR-two-dependent and type I IFN-dependent activity, NK cells were incubated with an anti-TLR2 Ab (T2.5), anti-IFNAR Ab (MMHAR-2), or a control Ab for 1 h before the addition of HCMV. Inhibition of endosomal maturation was achieved by treating NK cells with Bafilomycin A1 (25 nM; Sigma, Saint Louis, MO, USA) for 30 min before HCMV addition and during the 8 h of co-culture.

### Functional assays

Supernatants of non-treated or HCMV-exposed NK cells were collected after 24 and 48 h and frozen at −80°C. Thawed samples were assayed by ELISA for IFNγ and IFNα (Bender MedSystems, Vienna, Austria).

Natural killer cell degranulation was measured by the CD107a mobilization assay (Alter et al., 2004). NK cells were non-treated or exposed to HCMV TB40/E strain. After 24 h NK cells were harvested, washed and incubated with 1 μg anti-CD107a-FITC and 5 ng/ml monensin for 4 h at 37°C with 10% CO_2_, either alone or in the presence of autologous HCMV-infected monocyte-derived Dendritic Cells (moDC) or the K562 erythroleukemia cell line. P815 murine mastocytoma cell line was pre-incubated with anti-NKp46, anti-NKp30, and anti-CD16 mAb, and used as target in redirected degranulation assays. After co-culture, cells were stained with anti-CD56 mAb and analyzed by flow cytometry.

MoDCs were generated as described previously (Magri et al., 2011). After 5 days, cells were infected with HCMV TB40/E strain (moi 50–100). At 48 h post-infection DC were harvested and used as targets cells in degranulation assays. To monitor the infection rate, cells were washed twice and cytospin preparations were stained by indirect immunofluorescence with a mouse anti-CMV IE-1/IE-2 monoclonal antibody (clone mab810, Chemicon, Temecula, CA, USA).

### Measurement of mRNA levels

For RNA and cDNA preparation, NK cells (2 × 10^6^) were isolated by negative selection and exposed to TB40/E HCMV strain (moi 5–10) in combination with IL-12 (10 ng/ml) for 6 and 12 h. Cells were lysed in RLT buffer (300 μl, RNeasy system, QIAGEN, 74104) and total RNA was isolated using the same system. RNA was quantified in a NanoDrop^®^ (ND-1000 Spectrophotometer) and 250 ng were retro-transcribed to cDNA using SuperScript III reverse transcriptase and random primers (Invitrogen). For real-time quantitative PCR (RT-qPCR), LightCycler 480 SYBR Green I Master (Roche) and a Roche LightCycler 480 detection system (Roche) were used following the instructions provided by the manufacturers. Samples were normalized to GAPDH mRNA levels using the LightCycler 480 Software, version 1.5. Primer sequences for the PCR reactions were: 5′-GCC ATC AAT GAC CCC TTC ATT-3′ (Forward) and 5′-TTG ACG GTG CCA TGG AAT TT-3′ (Reverse) for GAPDH; 5′-AAA CTC ATG AGC AGT CTG CA-3′ (Forward) and 5′-AGG AGA TCT TCA GTT TCG GAG G-3′ (Reverse) for IFNβ (DeFilippis et al., 2010); 5′-GCA GGT CAT TCA GAT GTA GCG G-3′ (Forward) and 5′-TGT CTT CCT TGA TGG TCT CCA CAC-3′ (Reverse) for IFNγ (Janson et al., 2008).

### Statistical analysis

Statistical analysis was performed by the Student *t*-test using GraphPad Prism 5 software. Results were considered significant at the two-sided *P* level of 0.05.

## Results

### Direct recognition of HCMV by NK cells induces CD69 expression and synergizes with IL-12 in the production of IFNγ

**Figure 1:**
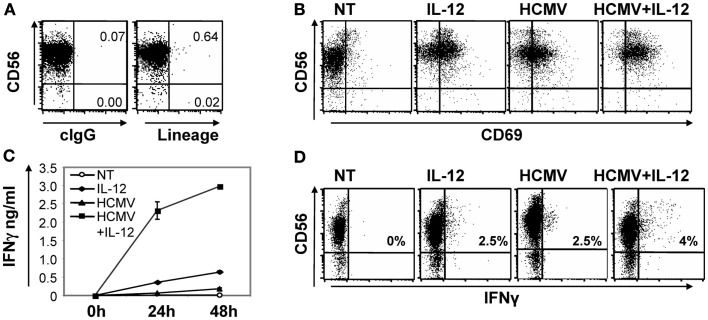
**Direct HCMV recognition induced CD69 expression in NK cells and enhanced IL-12-induced IFNγ secretion**. Purity of NK cells isolated by negative selection from peripheral blood of healthy donors was assessed by multicolor flow cytometry including anti-CD56-APC and anti-CD3-PE, -CD19-PE, -CD14-PE, -CD1a-PE, -CD123-PE as lineage exclusion cocktail **(A)**. Purified NK cells were left untreated (NT) or co-cultured with HCMV (MOI 5–10) for 24 and 48 h in the presence or absence of IL-12 (10 ng/ml) and IL-2 (200 μ/ml). At 24 h, CD69 expression on NK cells was tested by a double staining with anti-CD56-APC and CD69-PE specific mAbs by flow cytometry **(B)**. IFNγ was measured by ELISA in cell-free supernatants harvested at 24 and 48 h **(C)**. Intracellular IFNγ was monitored on NK cells co-cultured for 24 h with the different stimuli. Brefeldin A was added for the last 5 h and IFNγ staining was analyzed by flow cytometry. Inserts depict the proportion of IFNγ positive NK cells **(D)**. In all cases, data correspond to results from a representative donor out of at least three tested.

To assess whether NK cells could directly recognize HCMV we set up an experimental culture system including highly purified primary NK cells and viral particles of the TB40/E HCMV viral strain. Purity of the NK cells obtained was >98% as evaluated by flow cytometry including an anti-CD56 as an NK cell marker against a panel of mAbs including anti-CD19, -CD14, -CD3, -CD1a, and -CD123 as lineage exclusion cocktail (Figure 1A). In all experiments, NK cells were stimulated with 5–10 infective viral particles per cell (from now on referred as HCMV moi). We initially monitored the up-regulation of the early activation marker CD69 by flow cytometry and the production of IFNγ in cell-free supernatants by ELISA. IL-12 (10 ng/ml) was added to promote IFNγ production. After 24 h, HCMV and IL-12, alone or in combination, induced the expression of CD69 on NK cells (Figure 1B). HCMV-induced expression of CD69 was mostly restricted to CD56^dim^ NK cells, at variance with the general up-regulation induced by IL-12 treatment. On the other hand, incubation of NK cells with HCMV resulted in a modest production of IFNγ, but significantly enhanced the secretion induced by IL-12, as measured by ELISA in the cell-free supernatant after 24 and 48 h (Figure 1C).

Intracellular IFNγ staining showed that HCMV moderately increased the frequency of NK cells producing IFNγ in response to IL-12. The higher intensity of the IFNγ staining in NK cells treated with both stimuli indicated an enhancement or a more sustained IFNγ response per cell (Figure 1D). In contrast to the low IFNγ secretion detected by ELISA (Figure 1C), a proportion of the NK cells incubated with the virus contained intracellular IFNγ (Figure 1D). Differences in IFNγ production were not related to NK cell viability that was shown to be similar among the conditions tested, by AnnexinV/Propidium Iodide staining (data not shown).

**Figure 2:**
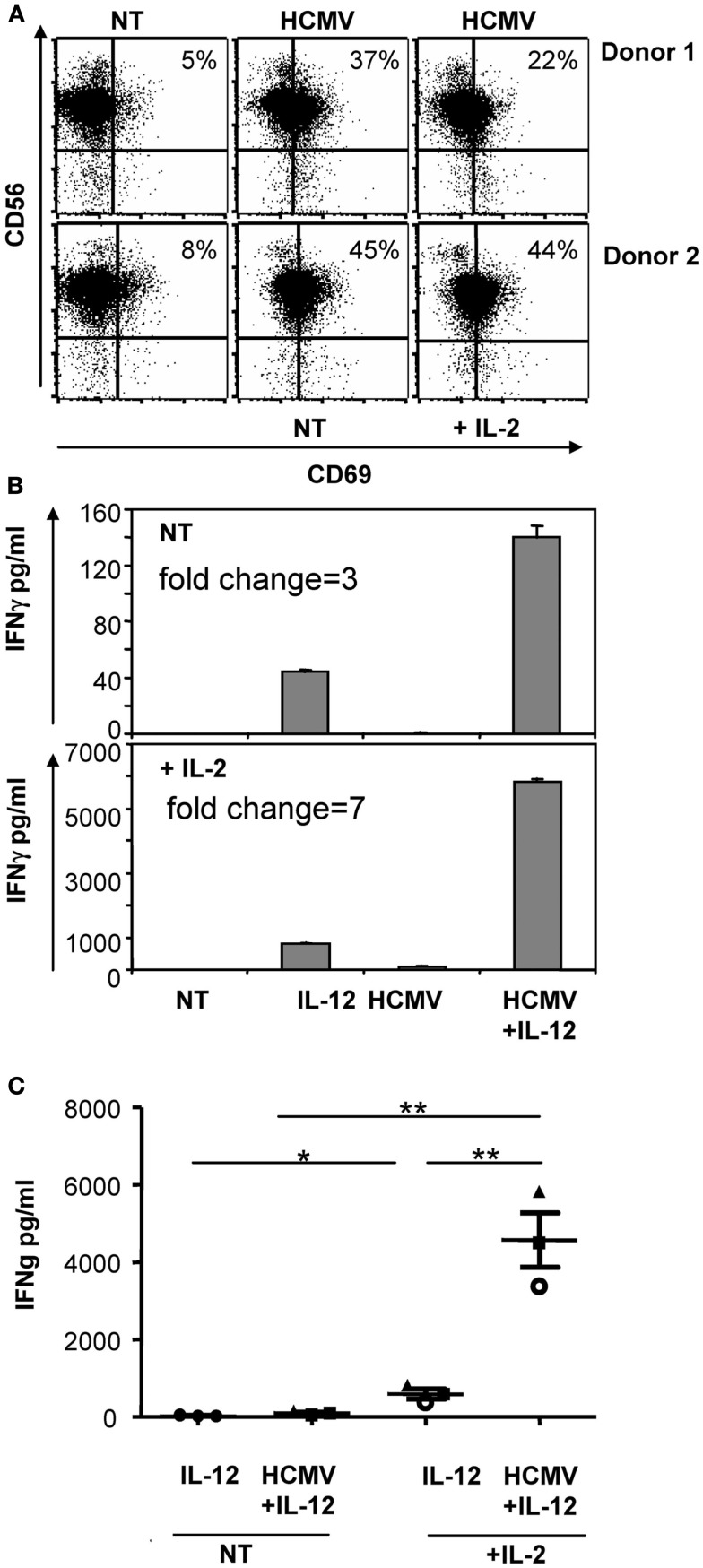
**IL-2 influence on the recognition of HCMV by NK cells**. Freshly purified NK cells were incubated with HCMV (MOI 5) and IL-12 (10 ng/ml) in the presence or absence of 200 μ/ml of IL-2. After 24 h, CD69 expression was analyzed by flow cytometry **(A)** and the secretion of IFNγ was monitored in cell-free supernatants by ELISA **(B,C)**. Bar graphs show the secretion of IFNγ of a representative experiment **(B)**. Scattered plot display results of the three donors independently tested **(C)**. Statistical analysis was performed by the Student *t*-test. Mean ± SEM are indicated (*p < 0.05; **p < 0.01).

Since previous experiments included IL-2 (200 μ/ml), we next assessed whether NK cells would equally respond to HCMV in the absence of IL-2. As shown in Figure 2, HCMV-induced CD69 up-regulation and enhanced IFNγ secretion independently of IL-2 signaling; nonetheless, IL-2 magnified the secretion of IFNγ in all conditions.

Overall, our data indicated that NK cells could directly recognize HCMV. Sensing of the virus promoted the expression of CD69 and enhanced the secretion of IFNγ in response to IL-12, regardless of IL-2 presence.

### Direct interaction with HCMV increases NK cell degranulation against HCMV-infected cells

**Figure 3:**
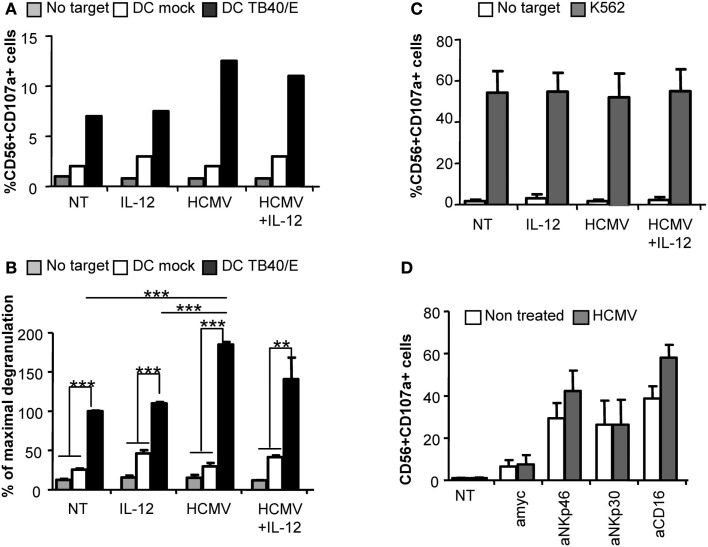
**Direct HCMV recognition enhances NK cell degranulation against HCMV-infected moDC**. Freshly purified NK cells were incubated alone (NT), or with IL-12 and/or HCMV TB40/E. After 24 h, cells were washed and the degranulation of NK cells was assessed after 5 h incubation with mock or HCMV TB40/E infected moDC **(A,B)**, and K562 **(C)** (E:T, 4:1). NK cell degranulation was monitored by CD107a staining analyzed by flow cytometry. **(A)** Bar graphs represent the percent of NK cells positive for CD107a in the absence of target cell (gray bars), in front of mock moDC (empty bars), or HCMV-infected moDC (black bars) from one representative donor. **(B)** Bar graph displaying the mean ± SEM of the normalized degranulation. Data in each experiment were normalized to the response of non-treated NK cells incubated with HCMV-infected DC (100%). Statistical analysis by the Student *t*-test (**p < 0.01; ***p < 0.0001) **(C)** Bar graph showing the mean ± SEM of the percent in degranulation to K562 of three donors tested **(D)** Bar graph displaying the mean ± SEM frequency of CD56+CD107a+ of non-treated (empty bars) or HCMV treated (gray bars) NK cells responding to P815 cells coupled to mAbs specific for NKp46 (Bab281), NKp30 (az20), CD16 (KD1), and anti-myc as control IgG in three independent experiments.

Degranulation assays were performed to evaluate whether direct recognition of HCMV would also influence NK cell cytotoxicity against HCMV-infected targets. Purified NK cells were incubated with HCMV in the presence or absence of IL-12. After 24 h, cells were harvested, washed to eliminate remaining viral particles and incubated with autologous moDC infected with HCMV as targets or the K562 human leukemia cell line as a positive control. The rate of moDC infection ranged between 40–60% depending on the donor. CD107a mobilization assays were used to monitor the proportion of NK cells secreting cytolytic granules, As shown in Figures 3A,B NK cells preincubated with HCMV displayed an increased degranulation against HCMV-infected moDC compared to non-treated NK cells, independently of IL-12, while showing similar response against K562 cells (Figure 3C). The NKp46 activating receptor has been previously shown to mediate the recognition of HCMV-infected myelomonocytic cells (Magri et al., 2011; Romo et al., 2011). In order to assess whether sensing of HCMV would modulate the activity of specific NK cell activating receptors, we set up P815-based redirected assays to analyze the degranulation triggered through NKp46, NKp30, and CD16. Purified NK cells pretreated with HCMV or untreated were confronted to P815 targets coupled to specific mAbs. As shown in Figure 3D, HCMV-primed NK cells tended to display an enhanced NKp46- and CD16-mediated degranulation in comparison to mock treated NK cells while comparably responding to NKp30 triggering.

Altogether, these results indicate that sensing of HCMV by NK cells facilitated their activation in response to HCMV-infected moDC.

### HCMV recognition activates several NK cell sensor mechanisms involving the production of type I IFNs and TLR2

**Figure 4:**
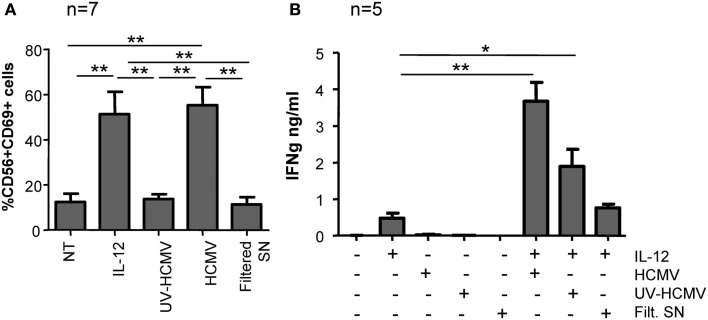
**NK cell activation in response to infective and UV-inactivated HCMV**. Freshly purified NK cells were incubated with infective-, UV-inactivated- HCMV, or filtered viral supernatant in the presence or absence of IL-12 (10 ng/ml). After 24 h, CD69 expression was analyzed by two-color flow cytometry with specific mAbs anti-CD69 and -CD56 **(A)** and IFNγ secretion was measured by ELISA in cell-free supernatants **(B)**. Bar graphs correspond to the mean ± SEM of the results obtained with the indicated donors. Statistic analysis by the Student *t*-test (*p < 0.05; **p < 0.01).

To further characterize the NK cell response to HCMV, CD69 expression was monitored in freshly purified NK cells cultured with infective-, UV-inactivated HCMV, and filtered viral supernatant for 24 h. Only infective but not UV-inactivated virus or supernatant devoid of viral particles induced CD69 expression on NK cells (Figure 4A). To assess IFNγ secretion, purified NK cells were stimulated in the same conditions in the presence of IL-12 (10 ng/ml). Enhanced secretion of IFNγ was mostly sustained by the infective form of HCMV, UV-inactivated virions showed a partial effect while the filtered viral supernatant did not significantly modify the amount of IFNγ secreted in response to IL-12 (Figure 4B). Thus, HCMV-induced NK cell activation was absolutely dependent on the presence of viral particles, ruling out the presence of other pathogen-associated molecular patterns or damage-associated molecular patterns in the virus stock supernatant.

**Figure 5:**
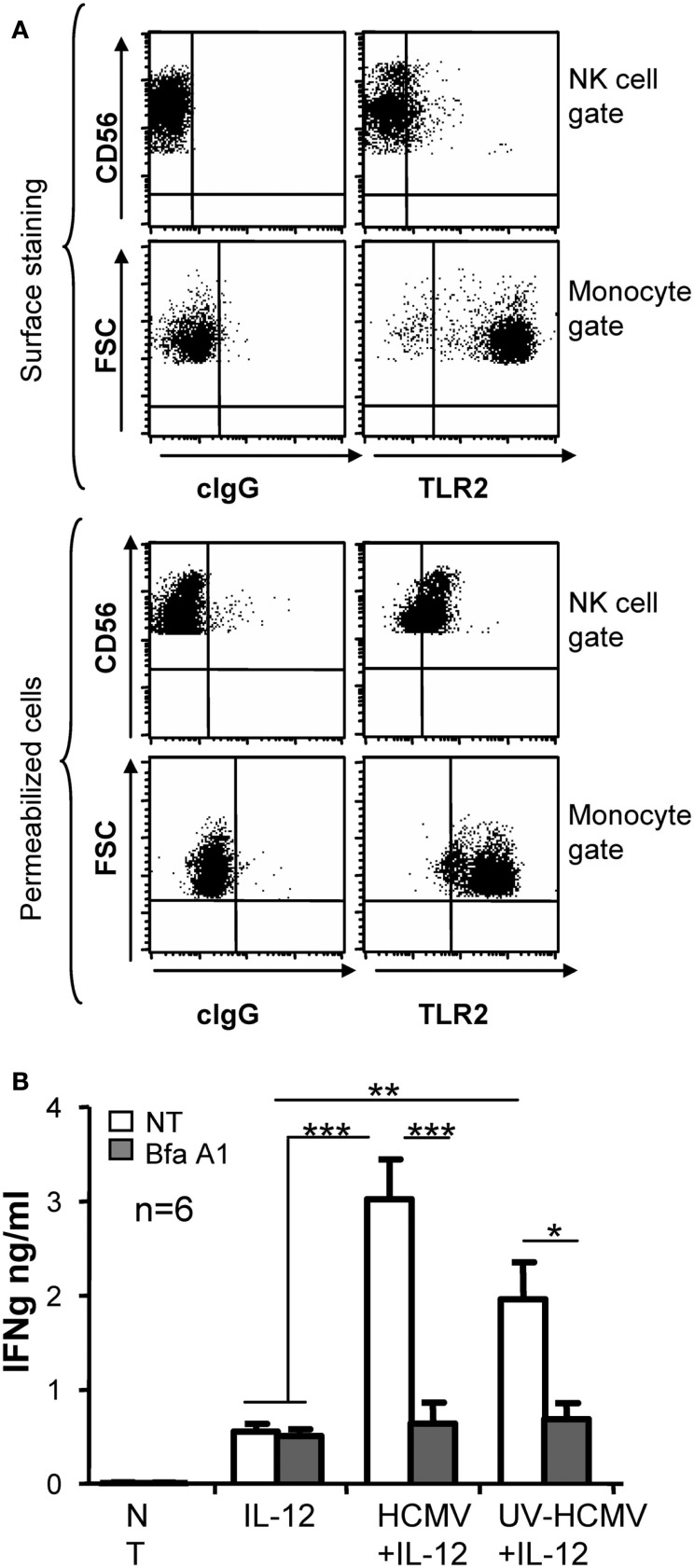
**Intracellular TLR2 localization and effect of Bafilomycin A1 treatment in HCMV sensing by NK cells**. PBMC samples were analyzed by three color flow cytometry with mAb specific for TLR2 or a control IgG in combination with anti-CD3-PerCP and anti-CD56-APC. For intracellular staining PBMC were fixed and permeabilized prior to the incubation with mAbs. Dot plots display the staining for TLR2 in the NK cell gate (CD56+CD3-) and in monocytes gated by forward and side light scatter. Data correspond to one representative donor out of three analyzed **(A)**. IFNγ secretion was measured by ELISA in supernatants from NK cells incubated with IL-12 and infective- or UV-inactivated HCMV, in the presence or absence of Bafilomycin A1 (Bfa A1, 25 nM). Empty and gray bars correspond to non-treated (NT) and Bfa A1 treated cultures. Bar graph showing the mean ± SEM of the data obtained from six donors **(B)**. Statistic analysis by the Student *t*-test (*p < 0.05; **p < 0.01; ***p < 0.0001).

The different response to infective and UV-inactivated virus suggested that HCMV triggered at least two distinct NK cell sensor mechanisms. Since TLR2 is one of the major HCMV sensors, we investigated whether it could be involved in the recognition of this virus by NK cells. Staining of freshly isolated PBMC with anti-TLR2 in combination with anti-CD56 and anti-CD3 showed that TLR2 expression was intracellular in NK cells, being barely detectable on the cell surface. In contrast, monocytes displayed TLR2 at the cell surface (Figure 5A). To test whether the endocytic pathway was involved in HCMV sensing, endosome maturation was blocked by adding bafilomycin A1, an inhibitor of the vacuolar-type H+-ATPase, along NK cell treatment with HCMV. Bafilomycin A1 prevented the increase in IFNγ triggered by both infective and Uv-inactivated virus while keeping unaltered the secretion in response to IL-12 (Figure 5B).

**Figure 6:**
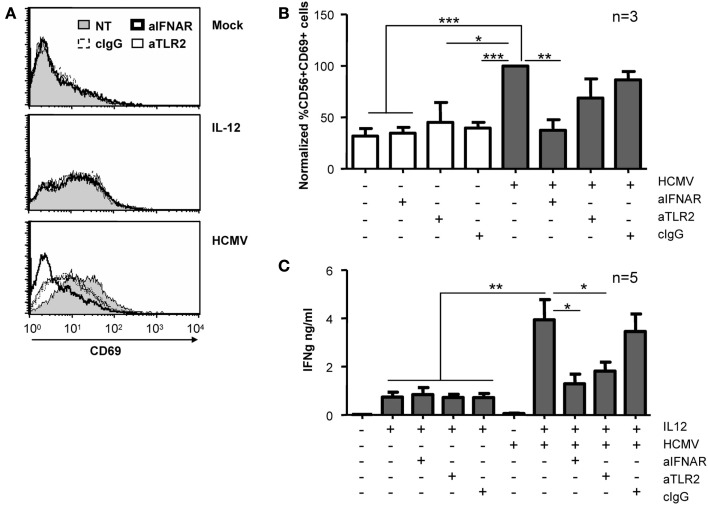
**Effect of IFNAR and TLR2 blockade on NK cell activation**. Freshly purified NK cells were left untreated or incubated with HCMV and IL-12 in the presence or absence of antagonizing antibodies for TLR2 and IFNAR. CD69 expression **(A,B)** and IFNγ secretion **(C)** were monitored at 24 h by flow cytometry and ELISA, respectively. **(A)** Histograms depict surface CD69 expression on NK cells in the absence of blocking mAbs (NT, gray profiles) or when cultures included anti-IFNAR (bold lines), anti-TLR2 (thin lines), or control IgG (dashed lines). Results from one representative donor are shown. **(B)** Bar graph showing the mean ± SEM of the normalized expression of CD69. Data in each experiment were normalized to the CD69 expressed by NK cells exposed to HCMV in the absence of blocking reagents (100%). **(C)** Bars graph displaying the mean ± SEM of IFNγ secretion in five donors. Statistic analysis by the Student *t*-test (*p < 0.05; **p < 0.01; ***p < 0.0001).

Given that type I IFNs have been previously shown to induce CD69 expression in NK cells (Waddell et al., 2010; Magri et al., 2011), we used blocking mAb for TLR-2 and IFNAR to test their participation in the NK cell response to HCMV. CD69 expression and the secretion of IFNγ were analyzed after treating NK cells with HCMV and IL-12 in the presence of blocking reagents. As shown in Figures 6A,B, HCMV-induced up-regulation of CD69 was completely prevented by blocking the type I IFN receptor, while the presence of TLR2 blocking mAb had a minor effect, comparable to control IgG. In contrast, HCMV-induced secretion of IFNγ was partially prevented by both anti-TLR2 and -IFNAR-blocking mAbs (Figure 6C). The expression of CD69 and the secretion of IFNγ in NK cells stimulated with IL-12 was comparable in the presence of blocking mAb (Figures 6A–C).

**Figure 7:**
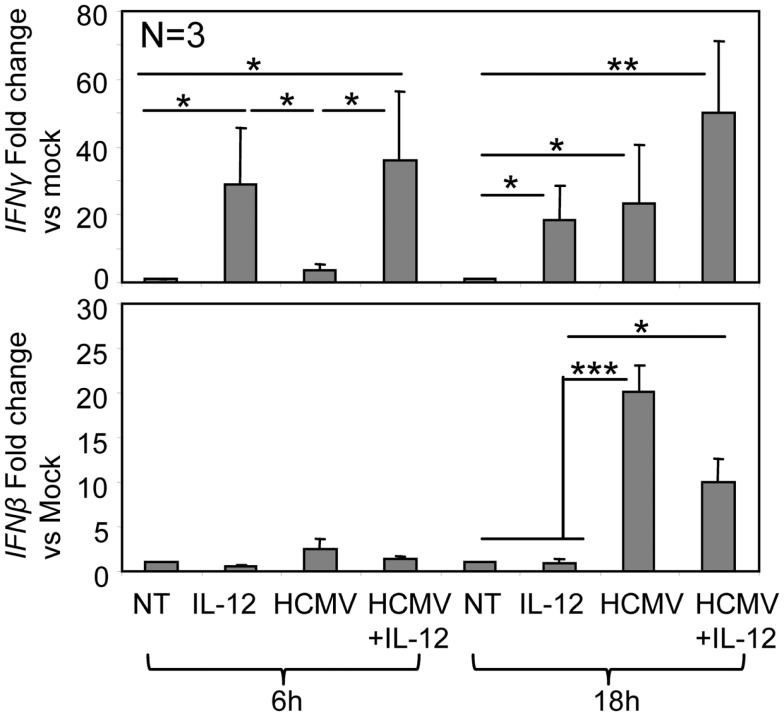
***IFN*γ and *IFN*β mRNA in NK cells along co-culture with HCMV**. Real-time PCR was performed on mRNA isolated from purified NK cells treated with IL-12 and HCMV TB40/E alone or in combination, for 6 and 18 h. Bar graphs indicate expression levels of *IFN*γ and *IFN*β transcripts. GAPDH was used to internally standardize the levels of gene expression. Data are expressed as fold difference between non-treated cells (whose levels of gene expression have been assumed as 1) and cells cultured with different stimuli. Bar graphs include results obtained with NK cells purified from three different donors (mean ± SD). Statistic analysis by the Student *t*-test (*p < 0.05; **p < 0.01; ***p < 0.0001).

The analysis of IFNγ and IFNβ mRNA in purified NK cells showed that IL-12 was initially driving the increase in IFNγ mRNA (6 h). After 18 h, HCMV enhanced IFNγ mRNA levels in both IL-12-treated and non-treated NK cells (Figure 7). HCMV but not IL-12 triggered IFNβ mRNA expression in NK cells, detected at 6 h and increasing after 18 h of treatment (Figure 7). IFNα was not detected in the supernatants (data not shown), pointing out the role of IFNβ in the observed type I IFN-dependent activation of NK cells.

Taken together, our results indicate that NK cell recognition of HCMV is mediated by at least two different sensor mechanisms. Infective HCMV triggers a type I IFN response, likely driven by autocrine IFNβ production, which induces CD69 surface expression, while TLR2 participates in the recognition of the viral particle promoting IFNγ secretion.

## Discussion

To date, the NK cell response to HCMV has been characterized in animal models or in *in vitro* culture systems that do not allow assessing whether the direct interaction between NK cells and HCMV may influence the anti-viral response. Given the fact that NK cells express functional PRRs, we developed an experimental system to examine the capacity of NK cells to sense HCMV. The data demonstrate that direct HCMV recognition results in TLR2 activation and the production of type I IFNs leading to the priming of NK cells as evidenced by (i) induced expression of the CD69 activation marker, (ii) enhanced secretion of IFNγ and, (iii) increased degranulation against HCMV-infected moDC.

The interaction of NK cells with HCMV triggered at least two sensor mechanisms distinguished by the response to infective and UV-inactivated viral particles. Recognition of infective HCMV-induced the expression of CD69 in CD56^dim^ NK cells. In agreement with previous reports (Waddell et al., 2010; Magri et al., 2011), IFNAR-blocking mAbs prevented the expression of this early activation marker, indicating that HCMV recognition by resting NK cells triggers the production of type I IFNs. The expression of IFNβ mRNA supported this cytokine as the likely candidate for the autocrine effect. Type I IFN responses in MCMV infection partially depend on TLR2 activation (Szomolanyi-Tsuda et al., 2006; Barbalat et al., 2009). In our experimental system, the inefficacy of anti-TLR2 mAb in preventing HCMV-induced CD69 up-regulation together with the failure of UV-inactivated virions to induce CD69 expression, argued against the involvement of any TLR in the type I IFN response of NK cells to HCMV. The requirement of infective virions for CD69 up-regulation suggests that HCMV may enter into NK cells, initiating its transcriptional program and thus, allowing the activation of cytosolic PRRs. Cellular receptors such as heparan sulfate proteoglycans and the β1-integrin VLA-6 found in resting NK cells (Perez-Villar et al., 1996) could, theoretically, mediate the entry of HCMV (Compton, 2004). Yet, according with the resistance of lymphocytes to HCMV productive infection, we did not detect nuclear accumulation of the immediate early HCMV proteins IE-1/IE-2 in NK cells co-cultured with infective virions (data not shown; Rice et al., 1984; Nowlin et al., 1991). Our results do not allow to pinpoint which cytosolic sensor/s are involved in the HCMV-dependent IFNβ response but likely candidates are Mda5 or RIG-1, reported to be expressed in NK cells and to induce IFNβ secretion in response to polyI:C, a synthetic analog to viral dsRNA (Duluc et al., 2009; Perrot et al., 2010). Whether any resting NK cell is susceptible to HCMV entry or whether a particular NK cell subset supports the IFNβ response to HCMV, deserves further attention.

On the other hand, simultaneous addition of IL-12 and HCMV to NK cells, promoted a substantial IFNγ secretion, exceeding the effect of IL-12 alone. In this case, antagonistic anti-TLR2 mAbs partially blocked the synergism, indicating the involvement of this TLR in the recognition of HCMV by NK cells. Indeed, HCMV envelope glycoproteins gB and gH bind TLR2, triggering NF-kB activation in other cell types (Boehme et al., 2006). The fact that UV-inactivated viral particles also enhanced IL-12-induced IFNγ secretion further supports the idea that structural virion component/s are being recognized by NK cells. Although, TLR2 was barely detected on the NK cell surface by flow cytometry, intracellular TLR2 showed a consistent staining, agreeing with a previous report (Eriksson et al., 2006). Consistent with the likely TLR2 location in NK cell endosomes, an inhibitor of endosomal maturation (i.e., Bafilomycin A1) prevented HCMV-dependent IFNγ secretion. As TLR2 recognizes hydrophobic PAMPs, it is possible that acidification of the endocytic pathway would allow hindered peptides in gB to become exposed and visible to TLR2. Considering the specialized role of NK cells in the control of viral infections, it is tempting to speculate that intracellular TLR2 could serve as a mechanism to ensure its activation in response to intracellular pathogens. In this line, TLR2 has also been involved in the recognition of hepatitis C virus, lymphocytic choriomeningitis virus, measles virus, vaccinia virus, herpes simplex virus, and *M. bovis* by NK cells and other cell types (Bieback et al., 2002; Chang et al., 2007; Zhu et al., 2007; Marcenaro et al., 2008; Martinez et al., 2010; Leoni et al., 2012).

Human Cytomegalovirus recognition by NK cells resulted in the priming of at least two important anti-viral effector mechanisms: IFNγ secretion and cytotoxicity against infected cells. Both TLR2 and type I IFNs enhanced the secretion of IFNγ, as indirectly shown by the partial blockade with antagonistic mAbs for TLR2 and IFNAR, respectively. Engagement of TLR2 may enhance IFNγ secretion through the activation of NF-kB and Ik-Bzeta, similarly to IL-18R activation (Akira et al., 2006). The late increase of HCMV-dependent IFNγ mRNA levels is reminiscent of the kinetics of IFNγ production upon IL-12+IL-18 stimulation in NK cells (Miyake et al., 2010; Kannan et al., 2011). Of note, two genetic studies have shown that specific TLR2 polymorphisms condition the probability to develop CMV disease in transplant recipients (Kijpittayarit et al., 2007; Kang et al., 2012). Assessing whether TLR2 polymorphisms could explain the important inter-individual variability in the magnitude of IFNγ secreted by NK cells in response to HCMV deserves attention. On the other hand, the partial blockade exerted by IFNAR-blocking mAb supports the participation of autocrine IFNβ in the secretion of IFNγ. Signaling through IFNAR could directly cooperate with IL-12 (Duluc et al., 2009) or indirectly support IFNγ secretion by promoting TLR expression (Miettinen et al., 2001).

Natural killer cell interaction with infective HCMV also facilitated the subsequent recognition and degranulation against infected targets. This effect was independent of IL-12 and presumably related to the autocrine effect of type I IFNs induced by the virus. The requirement for type I IFNs stimulation on NK cells for the development of efficient cytotoxic responses has been previously appreciated (Martinez et al., 2008), however, the underlying mechanism remains largely unaddressed. It is known that type I IFNs increase granzyme B, perforin, IFNγ, TNFα, and NKG2A mRNA levels (Mori et al., 1998; Martinez et al., 2008), suppressing two miRNA (miR-378 and miR30e) that negatively regulate perforin and granzyme B expression in resting NK cells (Wang et al., 2012). Redirected degranulation assays suggested that NK cell priming by HCMV enhanced NKp46-mediated activation, most probably participating in the enhanced NK cell degranulation against infected moDC, according to previous reports (Magri et al., 2011; Romo et al., 2011). Whether the increase in the activity of these particular activating receptors occurs as a consequence of type I IFN signaling or by an alternative pathway triggered by HCMV deserves further attention.

## Conclusion

Physiologically, NK cells present in the inflammatory infiltrates of HCMV active replication sites would have the chance of directly encounter viral particles. Our data shows that the direct recognition of a complex pathogen such as HCMV activates complementary PRRs leading to the autonomous priming of NK cell effector functions, a mechanism likely contributing to the anti-viral response.

## Conflict of Interest Statement

The authors declare that the research was conducted in the absence of any commercial or financial relationships that could be construed as a potential conflict of interest.
